# Maternal RSV vaccination for infant protection: A systematic review and meta‐analysis of phase 3 trials with an integrated economic evaluation

**DOI:** 10.1002/ijgo.70641

**Published:** 2025-11-04

**Authors:** Johnatan Torres‐Torres, Lourdes Rojas‐Zepeda, José Rafael Villafan‐Bernal, Raigam Martinez‐Portilla, Salvador Espino‐y‐Sosa, Pablo Cerda‐Flores, Elsa Romelia Moreno‐Verduzco, Irma Eloisa Monroy‐Muñoz, Ameyran Yolanda Gonzalez‐Guerrero, Juan Mario Solis‐Paredes, Javier Perez Duran

**Affiliations:** ^1^ Department of Reproductive and Perinatal Health Research Instituto Nacional de Perinatología Isidro Espinosa de los Reyes Mexico City Mexico; ^2^ Obstetric and Gynecology Department Hospital General de México “Dr Eduardo Liceaga” Mexico City Mexico; ^3^ Iberoamerican Research Network in Obstetrics Gynecology and Translational Medicine Mexico City Mexico; ^4^ Maternal‐Fetal Department Instituto Materno Infantil del Estado de Mexico Toluca Mexico; ^5^ Laboratory of Immunogenomics and Metabolic Diseases Instituto Nacional de Medicina Genomica Mexico City Mexico

**Keywords:** infant morbidity, maternal immunization, meta‐analysis, pregnancy, randomized controlled trials, respiratory syncytial virus, systematic review, vaccine safety

## Abstract

**Background:**

Respiratory syncytial virus (RSV) is a leading cause of hospitalization and mortality in early infancy. Maternal immunization offers a preventive strategy, but uncertainties regarding safety and economic value have limited its implementation.

**Objectives:**

To synthesize phase 3 randomized trial evidence on the efficacy and safety of maternal RSV vaccination and to estimate its potential population and economic impact. By integrating a scenario‐based modeling framework derived from pooled meta‐analytic estimates, this review provides high‐certainty, policy‐relevant evidence to guide maternal immunization strategies in diverse settings.

**Search Strategy:**

PubMed, MEDLINE, Scopus, and Google Scholar were searched up to April 2025 using predefined terms for RSV vaccination, pregnancy, and randomized controlled trials (RCTs).

**Selection Criteria:**

Phase 3 RCTs comparing maternal RSV vaccination with placebo and reporting neonatal or maternal outcomes were included. Non‐randomized studies, monoclonal antibody trials, or reports lacking extractable data were excluded.

**Data Collection and Analysis:**

Two reviewers independently screened studies, extracted data, and assessed risk of bias (RoB‐2). Pooled risk ratios (RR) and absolute risk differences were calculated with random‐effects models. Certainty of evidence was graded using Grading of Recommendations Assessment, Development and Evaluation (GRADE). A scenario‐based cost‐effectiveness model was applied to the Mexican birth cohort.

**Main Results:**

Four phase 3 RCTs (17 391 women) were included. Maternal RSV vaccination halved the risk of infant RSV infection (risk ratio [RR] 0.47, 95% confidence interval [CI]: 0.29–0.76; number needed to vaccinate [NNV] 85) and reduced severe disease by 64% (RR 0.36, 95% CI: 0.21–0.60; NNV 127). No increased risks were observed for preterm birth, pre‐eclampsia, or stillbirth. Certainty was moderate (any RSV) to high (severe RSV). In Mexico, universal vaccination at list price (US$295 per dose) would prevent approximately 20 769 infections and 228 neonatal deaths annually, though with high costs per case averted. At public‐sector pricing (US$50 per dose), cost‐effectiveness improved substantially.

**Conclusions:**

Maternal RSV vaccination is effective, safe, and potentially cost‐justifiable in high‐burden settings, supporting its integration into national immunization programs.

PROSPERO registration: CRD420251014636 (March 2025).

## 1 INTRODUCTION

Respiratory syncytial virus (RSV) is a leading cause of lower respiratory tract infections in infants, contributing significantly to global neonatal morbidity and mortality. According to the WHO, RSV is responsible for approximately 33 million cases of acute lower respiratory infections, 3.6 million hospitalizations, and 101 400 deaths in children under 5 years annually—with the highest burden observed in infants younger than 6 months of age.^1^ Given the absence of an effective antiviral treatment, preventive strategies are crucial in mitigating RSV‐related complications.

Maternal immunization has emerged as a promising approach to protect neonates through transplacental antibody transfer, providing passive immunity during the most vulnerable early months of life. This strategy is well established for influenza and pertussis vaccination during pregnancy and has recently been extended to RSV prevention. In 2023, the US Food and Drug Administration (FDA) approved the RSVpreF vaccine (Abrysvo, Pfizer) for use in pregnant women, marking a major milestone in RSV prevention.^2^ Similarly, the RSVPreF3‐Mat (GSK) has demonstrated efficacy in clinical trials.^3^ However, despite promising results, concerns regarding safety, particularly an increased risk of preterm birth in some studies, necessitate further evaluation.^4^, ^5^

Several randomized controlled trials (RCTs) have assessed the efficacy and safety of RSV vaccines administered during pregnancy, but findings remain heterogeneous. While some trials have reported high vaccine efficacy in preventing severe RSV‐related lower respiratory tract infections in neonates (up to 81.8% at 90 days postpartum),^6^ others have raised concerns regarding adverse pregnancy outcomes, particularly preterm birth.^3^ Given the public health implications of maternal RSV vaccination policies, a rigorous synthesis of phase 3 trial data is essential to provide definitive evidence on vaccine effectiveness and safety.

In addition to summarizing clinical efficacy and safety, contemporary vaccine evaluations increasingly integrate health impact and economic perspectives, particularly when informing public policy in low‐ and middle‐income settings. According to the WHO's *Guide for Evidence Synthesis and Health Technology Assessment for Immunization Programs* and the Grading of Recommendations Assessment, Development and Evaluation (GRADE) “Evidence‐to‐Decision” framework, evidence synthesis should extend beyond clinical outcomes to estimate population‐level and economic implications.^7^, ^8^, ^9^ Previous systematic reviews, including the recent Cochrane analysis, did not explore this translational dimension.^10^ Addressing this gap, our study incorporates a complementary modeling component—directly informed by the pooled meta‐analytic estimates—to contextualize the potential benefits and cost implications of maternal RSV vaccination for national immunization programs, illustrated through a case study in Mexico but with broader relevance to similar middle‐income settings.

This systematic review and meta‐analysis synthesize phase 3 randomized trial evidence to provide the most robust assessment of maternal RSV vaccination to date. It quantifies vaccine efficacy and safety across clinically relevant outcomes and extends these findings through a population‐ and cost‐impact modeling framework, translating pooled absolute effects into actionable evidence for maternal immunization policy.

## 2 MATERIALS AND METHODS

### 2.1 Protocol registration

This systematic review and meta‐analysis was prospectively registered in the International Prospective Register of Systematic Reviews (PROSPERO; registration ID CRD420251014636). Because the analysis relied exclusively on data extracted from previously published, peer‐reviewed articles, neither institutional ethics approval nor additional informed consent were required. The review was conducted and reported in full compliance with the PRISMA 2020 statement (Table S1). All included trials had obtained informed consent and ethical approval, as documented in their original publications.

### 2.2 Search strategy and information sources

A comprehensive search was conducted in PubMed, MEDLINE, Scopus, and Google Scholar to identify phase 3 randomized controlled trials (RCTs) evaluating the efficacy and safety of maternal RSV vaccination. The search strategy combined medical subject headings (MeSH) and free‐text keywords including: “Respiratory Syncytial Virus Vaccines,” “RSV vaccine,”, “maternal RSV immunization,” “vaccination in pregnancy,” and “pregnant women.” Boolean operators (AND, OR) were used to optimize sensitivity. The initial search was performed in March 2025 and updated in April 2025. Full details of the search strategy are provided in Table S2.

### 2.3 Eligibility criteria

Studies were eligible if they: (1) were phase 3 RCTs; (2) evaluated active RSV vaccination in pregnant women; (3) included a placebo control group; and (4) reported neonatal and/or maternal clinical outcomes. Studies focusing on monoclonal antibody therapies (e.g., nirsevimab), non‐randomized designs, case reports, preclinical studies, or secondary analyses without extractable outcome data were excluded.

### 2.4 Study selection and data extraction

Two reviewers independently screened titles and abstracts. Full texts of eligible studies were retrieved for assessment against predefined criteria. Disagreements were resolved through discussion or consultation with a third reviewer. If outcomes were presented only graphically, we used the R package digitize to extract numerical data. Authors were contacted for missing information when necessary. The PRISMA flow diagram summarizing the selection process is presented in Figure S1.

A structured data extraction form was used to collect study‐level and population‐level characteristics: author, publication year, geographic region, vaccine type, sample size, gestational age at vaccination, inclusion/exclusion criteria, neonatal outcomes (e.g., laboratory‐confirmed RSV infection, severe RSV disease, mortality), and maternal outcomes (e.g., pre‐eclampsia, preterm birth). Safety data were extracted for both intervention and control groups.

### 2.5 Risk of bias assessment

**FIGURE 1:**
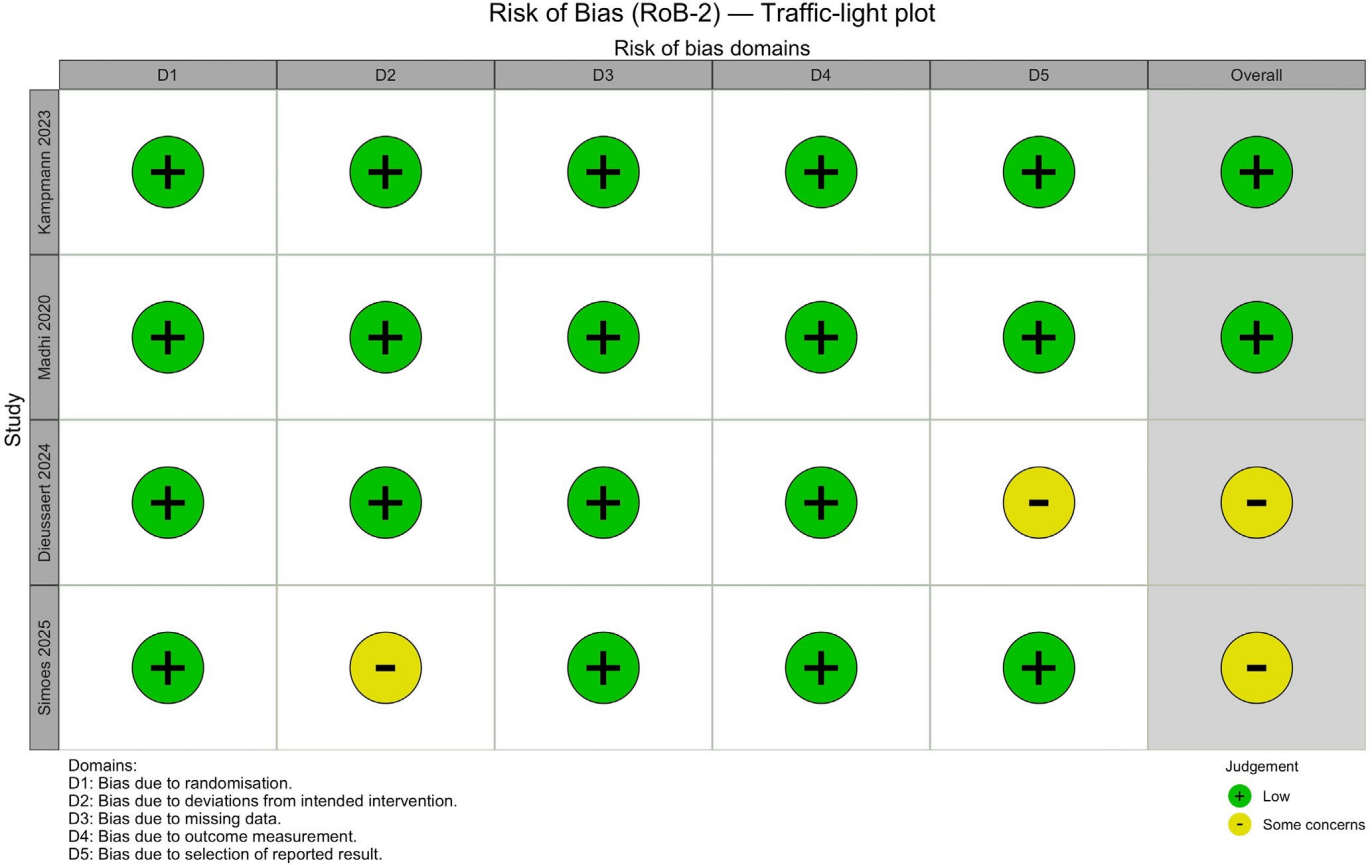
Review authors' judgments on each risk of bias domain for every included randomized controlled trial (risk of bias 2.0 [RoB‐2]).

The Cochrane risk of bias 2.0 (RoB‐2) tool was used to evaluate the methodological quality of the included RCTs. Two reviewers independently assessed risk of bias across five domains: randomization process, deviations from intended interventions, missing outcome data, measurement of outcomes, and selection of reported results. Any discrepancies were resolved by consensus. The results of the assessment are presented in Figure 1 and summarized in Table S3.

### 2.6 Statistical analysis

All meta‐analyses were conducted in R (version 4.3.0; RStudio interface) using the meta and metafor packages. For dichotomous outcomes, pooled risk ratios (RR) and risk differences (RD) were calculated with corresponding 95% confidence intervals (CI). Where the 95% CI of the RD did not cross zero, the number needed to vaccinate (NNV) was derived as the inverse of the absolute RD, with 95% CI calculated accordingly. Random‐effects models were fitted using the DerSimonian–Laird estimator for between‐study variance, and fixed‐effect models were also reported for comparison. Statistical heterogeneity was assessed with Cochran's Q test and quantified using the *I* ^2^ statistic, with values above 50% interpreted as substantial heterogeneity.

Publication bias was evaluated by visual inspection of funnel plot symmetry and by Egger's regression test, both implemented through meta and metafor.

### 2.7 Economic impact modeling

Following WHO guidance for vaccine evidence synthesis and impact modeling and GRADE Evidence‐to‐Decision principles, we extended the quantitative synthesis to estimate the population and economic implications of maternal RSV vaccination. The analysis used the pooled absolute risk reductions (risk differences) derived from the meta‐analysis to calculate the NNV for each outcome. These NNV values were applied to national demographic data to project the number of infections, severe cases, and neonatal deaths potentially averted under different coverage and pricing scenarios. Specifically, we used an annual live birth estimate of 1.89 million in Mexico, based on official population statistics.^11^

A deterministic, scenario‐based model was implemented in Microsoft Excel. The base‐case scenario assumed 100% vaccine coverage of the annual birth cohort and a per‐dose cost of US$295, corresponding to the current US list price of RSVpreF (Pfizer).^12^ The number of neonatal deaths averted was estimated by applying a conservative RSV case‐fatality rate of 1.1%, consistent with recent Latin American burden studies.^13^, ^14^, ^15^ Total program cost was calculated as the product of births and per‐dose price, while cost per event prevented was obtained by dividing total cost by the number of infections, severe cases, or deaths averted.

Sensitivity analyses explored lower vaccine prices (US$100 and US$50 per dose), reduced coverage levels (80% and 60%), and alternative case‐fatality assumptions (0.5%–1.5%). Indirect and long‐term benefits—such as hospitalization cost savings, prevention of chronic sequelae, and productivity gains—were intentionally excluded to maintain transparency and reproducibility within a static, first‐order deterministic framework. Although this simplified model does not incorporate probabilistic sensitivity analysis, dynamic transmission, or discounting, it provides a transparent and policy‐relevant approximation of the direct cost‐effectiveness of maternal RSV vaccination under realistic conditions in Mexico. This model was designed as a translational extension of the meta‐analysis, bridging pooled efficacy data with decision making relevance for countries evaluating maternal RSV immunization.

### 2.8 Certainty of evidence

The GRADE framework was used to assess the certainty of evidence for each outcome. GRADE domains included: risk of bias, inconsistency, indirectness, imprecision, and publication bias. The quality of evidence was categorized as high, moderate, low, or very low.

## 3 RESULTS

A total of 52 records were identified through database searches. After removing duplicates and screening titles and abstracts, 48 studies were excluded for irrelevance or duplication. Ultimately, four phase 3 randomized controlled trials were included in the meta‐analysis, encompassing 17 391 pregnant participants—10 475 in the vaccine groups and 6916 in the placebo groups. All trials evaluated laboratory‐confirmed RSV infection as the primary neonatal outcome and systematically reported adverse maternal and perinatal events.

Table 1 summarizes the key characteristics of the included studies. Trials were conducted between 2020 and 2024 across multiple geographic and demographic settings, with consistent eligibility criteria: healthy pregnant women aged 18–49 years, carrying singleton low‐risk pregnancies. The mean maternal age ranged from 26 to 29 years, and the gestational age at vaccination was between 30.8 and 32.0 weeks.

**TABLE 1:** General characteristics of the included studies.

Author, year and reference	Total n	N controls^a^	N vaccine^a^	Inclusion criteria	General characteristics of participants	WoG at vaccine administration	Vaccine type and doses	Follow‐up	Outcomes
Kampmann et al. (2023)^6^	7357	3676	3682	Healthy women, 49 years or younger and low risk healthy single pregnancies	Maternal age: 29 years Comorbidities:	30.8 (±3.5) w	120 μg of RSVpreF vaccine (60 μg each of RSV A and RSV B antigens)	1 year	Any RSV infection Severe RSV Hospitalization Adverse effects: cardiac disorders, infections, premature birth, pre‐eclampsia, gestational hypertension, fetal distress, neonatal death
Dieussaert et al. (2024)^3^	5328	1771	3557	Women aged 18–49 years, in good health, with a singleton fetus, and no known fetal genetic abnormalities	Maternal age: 29 years pBMI: 50.3% (18.5–24.9) 29% (25–29) 19.7 (≥30)	31.5 w	120 μg of RSVPreF3‐Mat	1 year	Any RSV infection Severe RSV Hospitalization Adverse effects: cardiac disorders, infections, premature birth, pre‐eclampsia, gestational hypertension, fetal distress, neonatal death
Simões et al. (2025)^16^	406	79	327	Women aged 18–49 years, in good health, with a singleton fetus, and no known fetal genetic abnormalities	Maternal age: 27	31.1 (±3.1) w	RSVpreF vaccine, 120 μg RSVpreF vaccine, 120 μg with aluminum hydroxide RSVpreF vaccine, 240 μg RSVpreF vaccine, 240 μg with aluminum hydroxide	1 year	Any RSV infection Severe RSV Hospitalization Adverse effects: cardiac disorders, infections, premature birth, pre‐eclampsia, gestational hypertension, fetal distress, and neonatal death
Madhi et al. (2020)^17^	4626	1581	3045	Women aged 18–40 years and low risk healthy single pregnancies	Maternal age: 26 pBMI: 28.55	32 (±2.6) w	120 μg of RSV fusion (F) nanoparticle	6 months	Any RSV infection Severe RSV Hospitalization Adverse effects: cardiac disorders, infections, premature birth, pre‐eclampsia, gestational hypertension, fetal distress, and neonatal death

*Note*: BMI, calculated as weight in kilograms divided by the square of height in meters.

Abbreviations: BMI, body mass index; pBMI, pre‐gestational body mass index; RSV, respiratory syncytial virus; WoG, weeks of gestation.

^a^ With complete follow‐up.

Two studies (Kampmann et al.,^6^ Dieussaert et al.^3^) administered a 120 μg dose of RSVpreF vaccine—formulated either as RSV A/B bivalent or as RSVPreF3‐Mat. Simões et al.^16^ tested four dosing regimens of RSVpreF with or without aluminum hydroxide adjuvant, and Madhi et al.^17^ evaluated a 120 μg nanoparticle formulation of the RSV fusion (F) protein. Follow‐up duration ranged from 6 months to 1 year postpartum. All studies reported neonatal RSV infection and severe RSV‐related illness, as well as adverse maternal and perinatal outcomes including preterm birth, pre‐eclampsia, gestational hypertension, fetal distress, and neonatal death.

### 3.1 Efficacy in preventing RSV infection in infants

**FIGURE 2:**
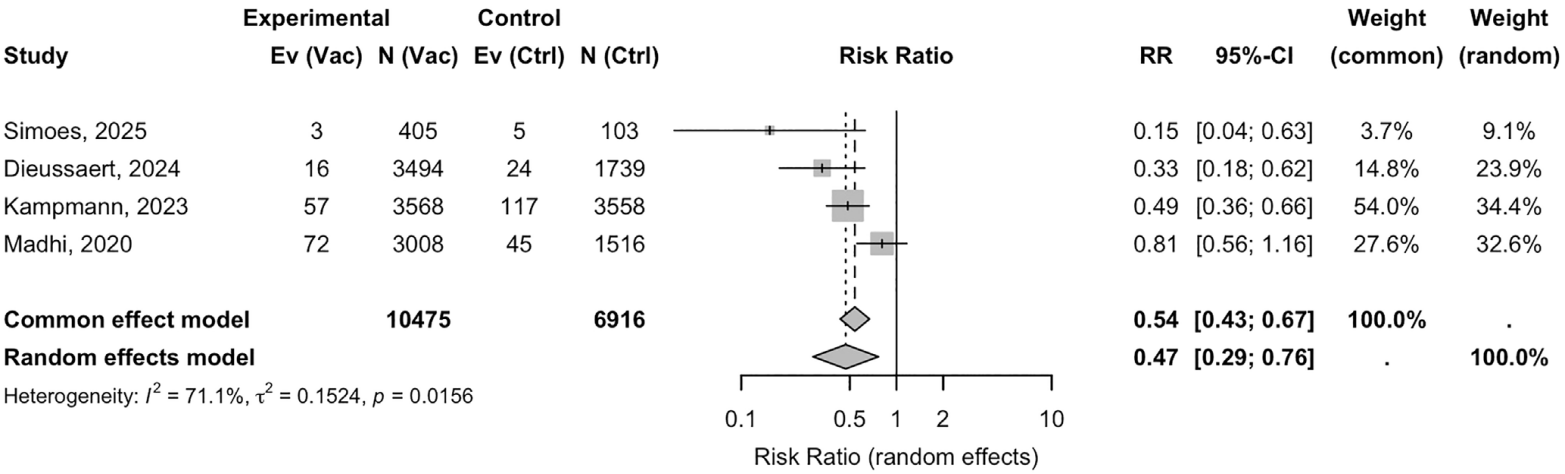
Random‐effects meta‐analysis of four phase 3 randomized controlled trials (RCTs): Maternal respiratory syncytial virus (RSV) vaccination reduces infant laboratory‐confirmed RSV infection up to 90–180 days (risk ratio [RR] 0.47; 95% confidence interval [CI]: 0.29–0.76; *I* ^2^ = 71.1%).

All four randomized controlled trials assessed the incidence of laboratory‐confirmed RSV infection in infants within the first 90–180 days of life. The pooled analysis demonstrated a statistically significant reduction in the risk of RSV infection among infants born to vaccinated mothers compared to placebo. Under the random effects model, maternal RSV vaccination was associated with a 53% relative reduction in risk (RR 0.47; 95% CI: 0.29–0.76; *P* = 0.015), despite moderate heterogeneity (*I* ^2^ = 71.1%) (Figure 2).

**FIGURE 3:**
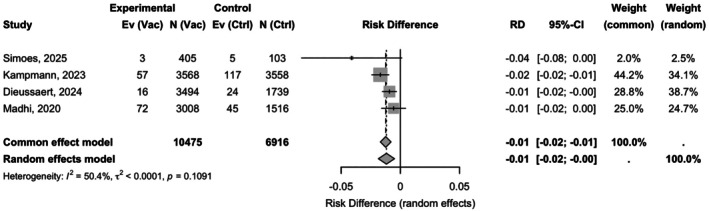
Random‐effects absolute risk difference for infant respiratory syncytial virus (RSV) infection at 90–180 days: Risk differences (RD) −0.01 (95% confidence interval [CI] −0.02 to −0.00; *I* ^2^ = 50.4%).

The absolute RD was −0.01 (95% CI: −0.02 to −0.00), corresponding to a NNV of 85 (95% CI: 54–201) to prevent one RSV infection in infants (Table S4). Heterogeneity for the RD model was lower (*I* ^2^ = 50.4%; *P* = 0.1091), suggesting greater consistency across studies for the absolute effect (Figure 3).

### 3.2 Protection against severe RSV disease

**FIGURE 4:**
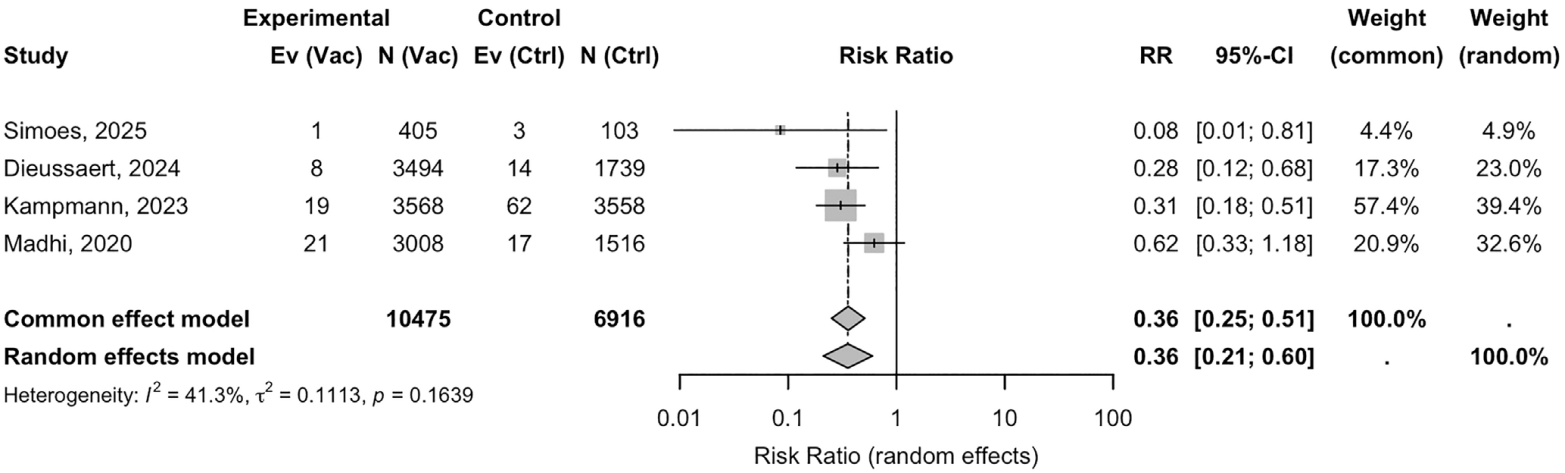
Random‐effects meta‐analysis of four phase 3 randomized controlled trials (RCTs): Maternal respiratory syncytial virus (RSV) vaccination lowers the risk of severe infant RSV disease (risk ratio [RR] 0.36; 95% confidence interval [CI]: 0.21–0.60; *I* ^2^ = 41.3%).

The pooled analysis under a random‐effects model revealed that maternal RSV vaccination was associated with a significant 64% relative reduction in the risk of severe RSV disease in infants (RR 0.36; 95% CI: 0.21–0.60; *P* < 0.0001), with moderate heterogeneity across studies (*I* ^2^ = 41.3%, *P* = 0.1639) (Figure 4).

**FIGURE 5:**
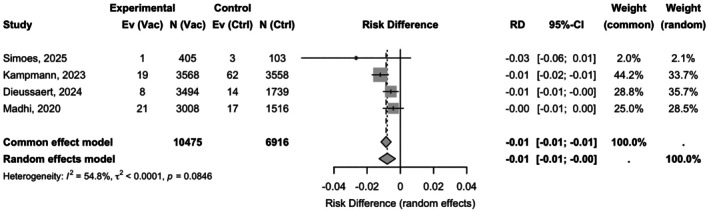
Random‐effects absolute risk difference for severe respiratory syncytial virus (RSV) disease at 90–180 days: Risk differences (RD) −0.01 (95% confidence interval [CI]: −0.01 to −0.00; *I* ^2^ = 54.8%).

The absolute RD was −0.01 (95% CI: −0.01 to −0.00), corresponding to a NNV of 127 (95% CI: 100–1000) to prevent one case of severe RSV‐related illness (Table S4). Consistency across studies was acceptable, with an *I* ^2^ of 54.8% for RD estimates and no significant heterogeneity (*P* = 0.0846) (Figure 5).

### 3.3 Maternal and infant safety outcomes

Across all four randomized controlled trials, maternal RSV vaccination was not associated with an increased risk of adverse outcomes during pregnancy, puerperium, or the neonatal period (Figure S2). As shown in Table 2, the pooled RR for preterm birth was 1.18 (95% CI: 0.84–1.66), and for pre‐eclampsia, 1.05 (95% CI: 0.86–1.28), indicating no statistically significant differences between vaccinated and control groups.

**TABLE 2:** Maternal and neonatal safety outcomes.

Outcome	Control risk (per 1000)	Vaccinated risk (per 1000; 95% CI)	Absolute difference (per 1000; 95% CI)	RR (95% CI)	Participants (studies)	*I* ^2^	Comments
Preterm birth	19	22 (16–32)	+3 (−3 to +13)	1.18 (0.84–1.63)	17 286 (4)	≤38%	No difference; CI crosses 1.0
Pre‐eclampsia	21	22 (18–27)	+1 (−3 to +6)	1.05 (0.86–1.28)	17 718 (4)	≈0%	No signal of harm; low event rates
Gestational hypertension	18	20 (16–24)	+2 (−2 to +6)	1.10 (0.89–1.36)	17 718 (4)	≈0%	Imprecision around the null
Adverse cardiac events	15	16 (12–20)	+1 (−2 to +6)	1.08 (0.85–1.39)	17 718 (4)	≈0%	Definitions heterogeneous across trials; absolute risks not illustrated
Stillbirth/fetal death	3	3 (2–5)	0 (−1 to +2)	0.91 (0.54–1.53)	17 286 (4)	≈0%	Very low incidence; no difference
Composite adverse effects (pregnancy, puerperium, perinatal)	159	167 (156–178)	+8 (−3 to +19)	1.05 (0.98–1.12)	17 718 (4)	≈0%	Small absolute difference; not significant

Abbreviations: CI, confidence interval; RR, risk ratio.

Similarly, stillbirth rates were comparable (RR 0.91; 95% CI: 0.54–1.53), with no evidence of increased risk. In the analysis of gestational hypertension, the pooled RR was 1.10 (95% CI: 0.89–1.36), and for adverse cardiac events, 1.08 (95% CI: 0.85–1.39), both suggesting no significant association with maternal RSV immunization.

Finally, the risk of composite adverse effects during pregnancy, puerperium, and perinatal conditions remained unchanged between groups (RR 1.05; 95% CI: 0.98–1.12). Across all safety outcomes, statistical heterogeneity was uniformly low or absent (*I* ^2^ ranging from 0% to 37.8%), supporting the consistency and reliability of these findings across trials and populations.

### 3.4 Consistency and publication bias

Sequential exclusion of individual studies did not materially alter the pooled estimates. None of the included trials was discontinued early due to safety concerns. Funnel plot symmetry and Egger's regression test suggested no evidence of publication bias for either the primary (any RSV infection, *P* = 0.42) or secondary (severe RSV infection, *P* = 0.37) outcomes (Figure S3).

### 3.5 Certainty of the evidence

Using the GRADE approach, the certainty of evidence for the primary efficacy outcome (any RSV infection) was rated as moderate, downgraded for imprecision due to wide confidence intervals in one trial. Evidence for severe RSV disease prevention was rated as high, supported by narrow confidence intervals and consistent findings. Certainty for maternal and neonatal safety outcomes ranged from moderate to high, with downgrading applied selectively due to imprecision or low event rates. The full GRADE assessments are presented in Table S5.

### 3.6 Projected cost‐effectiveness of maternal RSV vaccination in Mexico

To evaluate the economic impact of implementing maternal RSV immunization at the national level in Mexico, we conducted a scenario‐based cost‐effectiveness analysis informed by pooled estimates from the meta‐analysis and national demographic data. The base case assumed 100% coverage and a per‐dose cost of US$295^12^ (current US list price for RSVpreF). Under this scenario, the total annual investment would be US$557.55 million, preventing an estimated 20 769 RSV infections, 14 882 severe cases, and 228 neonatal deaths (case‐fatality rate 1.1%).^13^, ^14^, ^15^ This corresponds to US$26845 per infection averted, US$37465 per severe case averted, and US$2440455 per neonatal death averted.

Sensitivity analyses explored reduced pricing, partial coverage, and alternative fatality rates. At a public‐sector price of US$50/dose, the cost per infection and per severe case averted fall to US$4550 and US$6350, respectively, with a cost per death averted of US$413636. Relative to Mexico's value of a statistical life (VSL) (≈US$150000–US$350000),^18^ this level approaches the upper bound (~1.18× the US$350000 benchmark), and further price reductions would bring it within the range. Varying the case‐fatality rate to 1.5% or 0.5% yields US$1789667 and US$5369000 per death averted, respectively. Coverage reductions (80% and 60%) lower total health impact and program spend proportionally but do not change unit costs per case averted.

While cost per case prevented exceeds generic WHO cost‐effectiveness thresholds (1–3× GDP per capita; ~US$11000–US$33000),^19^ these thresholds apply to cost per DALY/QALY, not to cost per case; comparisons should therefore be interpreted with caution. Moreover, our estimates are conservative and exclude indirect and long‐term benefits (fewer hospitalizations and pediatric intensive care unit (ICU) admissions, prevention of chronic respiratory sequelae, caregiver productivity gains, and reduced antimicrobial exposure) (Table 3).

**TABLE 3:** Scenario‐based cost‐effectiveness of maternal RSV vaccination in Mexico.

Coverage	Price per dose (USD)	Total cost (USD)	Infections prevented	Severe cases prevented	Case fatality rate	Deaths averted	Cost per infection averted (USD)	Cost per severe case averted (USD)	Cost per death averted (USD)	Relative to Mexico VSL^a^
100%	$295	$557 550 000	20 769	14 882	1.1%	228	$26 845	$37 465	$2 440 455	≈7.0× upper VSL
100%	$100	$189 000 000	20 769	14 882	1.1%	228	$9100	$12 700	$827 273	≈2.36× upper VSL
100%	$50	$94 500 000	20 769	14 882	1.1%	228	$4550	$6350	$413 636	≈1.18× upper VSL
80%	$295	$446 040 000	16 615	11 906	1.1%	183	$26 845	$37 465	$2 440 455	≈7.0× upper VSL
60%	$295	$334 530 000	12 462	8929	1.1%	137	$26 845	$37 465	$2 440 455	≈7.0× upper VSL
100%	$295	$557 550 000	20 769	14 882	1.5%	312	$26 845	$37 465	$1 789 667	≈5.11× upper VSL
100%	$295	$557 550 000	20 769	14 882	0.5%	104	$26 845	$37 465	$5 369 000	≈15.34× upper VSL

*Note*: National birth cohort = 1.89 million. Infections and severe cases prevented derived from pooled NNV (91 and 127). Deaths averted = infections prevented × CFR. Static, deterministic model; no discounting or indirect effects. USD values rounded to nearest dollar.

Abbreviations: NNV, number need to vaccinate; RSV, respiratory syncytial virus; VSL, value of a statistical life.

^a^ VSL comparator shown for mortality only. Upper‐bound VSL used here = US$350000 (published range ~ US$150000–US$350000). “≈k×” denotes the ratio cost per death averted/350000.

## 4 DISCUSSION

### 4.1 Principal findings

In this phase 3–only systematic review and meta‐analysis (17 391 pregnant participants), maternal RSV vaccination reduced any infant RSV infection (RR 0.47; 95% CI: 0.29–0.76; NNV 85) and severe RSV disease (RR 0.36; 95% CI: 0.21–0.60; NNV 127) during the first 90–180 days of life. No increased risks were observed for preterm birth (RR 1.18), pre‐eclampsia (1.05), stillbirth (0.91), or other maternal–perinatal outcomes. Certainty ranged from moderate (any RSV) to high (severe RSV), supporting the robustness of the evidence.

### 4.2 Comparison with existing literature

Our findings align with prior systematic reviews, including a recent Cochrane analysis that confirmed the efficacy of RSVpreF in reducing medically attended RSV illness.^10^ The present analysis extends the literature by: (1) restricting to phase 3 RCTs updated through 2025, (2) quantifying absolute effects (NNV and risk differences) alongside relative effects, (3) providing a comprehensive safety profile using RoB‐2 and GRADE and (4) adding a scenario‐based economic evaluation tailored to Mexico. Together, these features increase external validity for policy decisions in Latin America.

Across trials, safety signals were consistently null for maternal and neonatal outcomes, with low heterogeneity (*I* ^2^ 0%–37%) in pooled safety estimates. One trial (RSVPreF3‐Mat, GSK) reported an imbalance in preterm birth and stopped early; however, the pooled estimate does not confirm an increased risk, and the domain was adjudicated as “some concerns” in RoB‐2 and downgraded in GRADE where applicable. Heterogeneity was higher for “any RSV infection” (*I* ^2^ ≈ 71%) than for severe disease (≈41%). This is expected given differences in case definitions and follow‐up windows (90 vs. 180 days); importantly, the direction of effect remained protective across studies.

This evidence is particularly relevant to Latin America, where the RSV burden remains high. In Mexico, respiratory infections accounted for over 1500 deaths in children under five in 2023, with infants under 1 year comprising the majority.^20^ In Argentina, RSV is responsible for nearly one‐third of lower respiratory tract infections in children and contributes to up to 22% of infant mortality.^14^ Based on national birth rates and the pooled effect sizes from this analysis, a base‐case Mexican program (100% coverage; US$295/dose) could prevent ~20 769 infections, ~14 882 severe cases, and ~ 228 neonatal deaths per year, highlighting the potential for meaningful population‐level impact. This clinical impact is further underscored by the substantial burden RSV imposes on healthcare systems across the region. In Mexico, lower respiratory tract infections are among the most common causes of hospitalization in children under five, with the highest incidence occurring in infants younger than 6 months.^21^ Similarly, in Argentina, respiratory syncytial virus is a principal driver of hospital admissions during the winter season, with increased risk and mortality in infants with comorbidities.^22^

Beyond clinical outcomes, this study is among the first to model the economic viability of maternal RSV vaccination in a Latin American setting using real‐world data and vaccine pricing. Assuming a per‐dose cost of US$295 and full national coverage, the projected cost per infection averted is US$26845, and US$37465 per severe case. When contextualized using two standard economic benchmarks—the VSL in Mexico (US$150000–US$350000) and the WHO‐recommended willingness‐to‐pay threshold (1–3× GDP per capita; ~US$11000–US$33000)—these values suggest that maternal RSV vaccination may be cost‐beneficial, especially when considering the cost per death averted (~US$2.44 million, based on a conservative 1.1% case‐fatality rate). Importantly, these calculations are conservative and exclude indirect and long‐term benefits. These include reductions in hospitalization and pediatric ICU use, prevention of chronic respiratory sequelae, increased parental productivity, reduced antibiotic exposure, and lower risk of antimicrobial resistance. Real‐world studies from Argentina estimate average hospitalization costs of approximately US$587.79 per RSV case, increasing to >US$1500 for cases requiring pediatric intensive care.^23^ In Chile, RSV‐related hospitalizations cost US$632 for low‐risk and US$1137 for high‐risk infants, with statistically significant cost differences.^24^ Additionally, neonates with healthcare‐acquired RSV infection incur hospital costs that are on average 41.9% higher, with total direct costs exceeding those of non‐RSV respiratory infections by >200%.^23^ At public‐sector pricing (e.g., US$50/dose), the cost per death averted falls to $413 636, with markedly lower costs per infection and severe case averted figures that strengthen the economic case for implementation within antenatal care.

This integrative approach contrasts with previous systematic reviews, including the recent Cochrane analysis, which focused exclusively on clinical efficacy and safety endpoints. By incorporating a scenario‐based economic model directly derived from meta‐analytic effect sizes, our study bridges the gap between evidence synthesis and implementation policy, providing a framework that can be adapted for vaccine introduction decisions across diverse health systems, particularly in low‐ and middle‐income settings, contributing to global respiratory disease prevention.

### 4.3 Strengths and limitations

Strengths include an exclusive focus on phase 3 RCTs, adherence to PRISMA, dual RoB‐2 assessment, GRADE by outcome, and concurrent estimation of relative and absolute effects. The economic component translates effect sizes into policy‐relevant scenarios. Limitations include the limited number of trials, variability in infection case definitions and follow‐up windows (contributing to heterogeneity for the “any RSV” endpoint), and a static economic model without indirect effects or long‐term cost offsets. Pricing and coverage are uncertain and will depend on procurement and implementation strategies; real‐world effectiveness and program costs may differ due to seasonality and health‐system capacity. Beyond these considerations, this study offers a replicable framework that integrates meta‐analytic and economic modeling approaches for future vaccine evaluations and policy analyses.

### 4.4 Implications

Health systems in high‐incidence settings should consider maternal RSV vaccination as part of antenatal services, particularly if public‐sector pricing is attainable. Priorities for research include probabilistic sensitivity analyses, price‐threshold (break‐even) estimates, incorporation of hospitalization/ICU costs and long‐term respiratory sequelae, and equity‐focused implementation studies (coverage among adolescents, rural populations, and those with limited access to neonatal intensive care). Active pharmacovigilance should monitor preterm birth and other rare outcomes as roll‐out expands.

## 5 CONCLUSION

This systematic review and meta‐analysis of phase 3 trials provide the strongest available evidence that maternal RSV vaccination is both effective and safe. Immunization during pregnancy significantly reduces neonatal RSV infection and severe disease, with no increase in adverse maternal or perinatal outcomes. These findings are particularly relevant for low‐ and middle‐income countries, where RSV is a major cause of infant morbidity and mortality and access to neonatal intensive care is limited. With a favorable number needed to vaccinate, moderate‐to‐high certainty of evidence, and cost‐effectiveness approaching accepted thresholds at public‐sector pricing, maternal RSV vaccination emerges as a clinically impactful and economically justifiable intervention. Its integration into antenatal care offers a scalable opportunity to reduce inequities in respiratory protection for the most vulnerable newborn populations and provides a solid foundation for its inclusion in national immunization programs and global maternal–child health policies.

## AUTHOR CONTRIBUTIONS

Johnatan Torres‐Torres (JTT) and Raigam Martinez‐Portilla (RMP) conceived the study. JTT, Lourdes Rojas‐Zepeda (LRZ), Pablo Cerda‐Flores (PCF), and José Rafael Villafan‐Bernal (JRVB) designed the methodology and protocol. The systematic search and study selection were conducted by LRZ, PCF, Irma Eloisa Monroy‐Muñoz (IEMM), Ameyran Yolanda González‐Guerrero (AYGG), and Juan Mario Solís‐Paredes (JMSP). Data curation was performed by LRZ, IEMM, AYGG, and Javier Pérez Duran (JPD). RoB‐2 and GRADE assessments were undertaken by JTT, JRVB, and Elsa Romelia Moreno‐Verduzco (ERMV). Formal analysis, including meta‐analysis and economic modeling, was carried out by JTT and JRVB, who also generated the visualizations (forest, funnel, and risk‐of‐bias plots). JTT drafted the original manuscript. Critical review and editing were provided by RMP, Salvador Espino‐y‐Sosa (SEyS), LRZ, ERMV, IEMM, AYGG, JMSP, JPD, and JRVB. Supervision was provided by RMP and SEyS. Project administration was managed by JTT and SEyS. JTT and RVB served as guarantors of the work.

## CONFLICT OF INTEREST STATEMENT

The authors declare that they have no conflicts of interest.

## PROTOCOL AND REGISTRATION

The review protocol is registered with PROSPERO (registration ID CRD420251014636); URL: https://www.crd.york.ac.uk/PROSPERO/view/CRD420251014636.

## Supporting information


Data S1.

## Data Availability

The datasets generated and analyzed during this study are available from the corresponding author upon reasonable request. Data access will be granted to researchers with a justified proposal for scientific use. Requests should be directed to: torresmmf@gmail.com.
