# Supplementary material for: Maternal RSV vaccination for infant protection: A systematic review and meta‐analysis of phase 3 trials with an integrated economic evaluation
Source: Int J Gynaecol Obstet. 2025 Nov 4;173(1):63–73. doi: 10.1002/ijgo.70641 (PMC12988398; doi:10.1002/ijgo.70641)
Supplement: Supplementary file 1 — Data S1. [file IJGO-173-63-s001.pdf]

PRISMA

PRISMA 2020 Checklist

Table S1. PRISMA Checklist

Section and Topic	Item #	Checklist item	Location where item is reported
**TITLE**			
Title	1	Identify the report as a systematic review.	Title page
**ABSTRACT**			
Abstract	2	See the PRISMA 2020 for Abstracts checklist.	Structured Abstract
**INTRODUCTION**			
Rationale	3	Describe the rationale for the review in the context of existing knowledge.	Introduction, paragraphs 1–3
Objectives	4	Provide an explicit statement of the objective(s) or question(s) the review addresses.	Introduction, final paragraph
**METHODS**			
Eligibility criteria	5	Specify the inclusion and exclusion criteria for the review and how studies were grouped for the syntheses.	Methods – Eligibility criteria
Information sources	6	Specify all databases, registers, websites, organisations, reference lists and other sources searched or consulted to identify studies. Specify the date when each source was last searched or consulted.	Methods – Search strategy and information sources; Table S1
Search strategy	7	Present the full search strategies for all databases, registers and websites, including any filters and limits used.	Table S1 (Supplementary Material)
Selection process	8	Specify the methods used to decide whether a study met the inclusion criteria of the review, including how many reviewers screened each record and each report retrieved, whether they worked independently, and if applicable, details of automation tools used in the process.	Methods – Study selection
Data collection process	9	Specify the methods used to collect data from reports, including how many reviewers collected data from each report, whether they worked independently, any processes for obtaining or confirming data from study investigators, and if applicable, details of automation tools used in the process.	Methods – Data extraction
Data items	10a	List and define all outcomes for which data were sought. Specify whether all results that were compatible with each outcome domain in each study were sought (e.g. for all measures, time points, analyses), and if not, the methods used to decide which results to collect.	Methods – Outcomes
	10b	List and define all other variables for which data were sought (e.g. participant and intervention characteristics, funding sources). Describe any assumptions made about any missing or unclear information.	Methods – Data extraction
Study risk of bias assessment	11	Specify the methods used to assess risk of bias in the included studies, including details of the tool(s) used, how many reviewers assessed each study and whether they worked independently, and if applicable, details of automation tools used in the process.	Methods – Risk of bias assessment; Table S2
Effect measures	12	Specify for each outcome the effect measure(s) (e.g. risk ratio, mean difference) used in the synthesis or presentation of results.	Methods – Statistical analysis
Synthesis	13a	Describe the processes used to decide which studies were eligible for each synthesis (e.g. tabulating the study intervention characteristics	Methods –

Table S1. PRISMA Checklist			
**Section and Topic**	**Item #**	**Checklist item**	**Location where item is reported**
methods		and comparing against the planned groups for each synthesis (item #5)).	Statistical analysis
	13b	Describe any methods required to prepare the data for presentation or synthesis, such as handling of missing summary statistics, or data conversions.	Methods – Statistical analysis
	13c	Describe any methods used to tabulate or visually display results of individual studies and syntheses.	Figures 1–4, Table 2; Figure S1
	13d	Describe any methods used to synthesize results and provide a rationale for the choice(s). If meta-analysis was performed, describe the model(s), method(s) to identify the presence and extent of statistical heterogeneity, and software package(s) used.	Methods – Statistical analysis
	13e	Describe any methods used to explore possible causes of heterogeneity among study results (e.g. subgroup analysis, meta-regression).	Methods – Statistical analysis
	13f	Describe any sensitivity analyses conducted to assess robustness of the synthesized results.	Methods – Statistical analysis
Reporting bias assessment	14	Describe any methods used to assess risk of bias due to missing results in a synthesis (arising from reporting biases).	Methods – Statistical analysis; Funnel plots (Figure S2)
Certainty assessment	15	Describe any methods used to assess certainty (or confidence) in the body of evidence for an outcome.	Methods – Certainty of evidence (GRADE); Table S3
**RESULTS**			
Study selection	16a	Describe the results of the search and selection process, from the number of records identified in the search to the number of studies included in the review, ideally using a flow diagram.	Results – Study selection; PRISMA flow diagram
	16b	Cite studies that might appear to meet the inclusion criteria, but which were excluded, and explain why they were excluded.	Results – Study selection
Study characteristics	17	Cite each included study and present its characteristics.	Results – Study characteristics; Table 1
Risk of bias in studies	18	Present assessments of risk of bias for each included study.	Results – Risk of bias; Table S2

Table S1. PRISMA Checklist

Section and Topic	Item #	Checklist item	Location where item is reported
Results of individual studies	19	For all outcomes, present, for each study: (a) summary statistics for each group (where appropriate) and (b) an effect estimate and its precision (e.g. confidence/credible interval), ideally using structured tables or plots.	Results – Figures 1–4, Table 2
Results of syntheses	20a	For each synthesis, briefly summarise the characteristics and risk of bias among contributing studies.	Results – Study characteristics and RoB
	20b	Present results of all statistical syntheses conducted. If meta-analysis was done, present for each the summary estimate and its precision (e.g. confidence/credible interval) and measures of statistical heterogeneity. If comparing groups, describe the direction of the effect.	Results – Figures 1–4, Table 2
	20c	Present results of all investigations of possible causes of heterogeneity among study results.	Results – Heterogeneity statistics (I^2, p-values)
	20d	Present results of all sensitivity analyses conducted to assess the robustness of the synthesized results.	Results – Consistency and publication bias
Reporting biases	21	Present assessments of risk of bias due to missing results (arising from reporting biases) for each synthesis assessed.	Results – Consistency and publication bias; Figure S2
Certainty of evidence	22	Present assessments of certainty (or confidence) in the body of evidence for each outcome assessed.	Results – Certainty of evidence; Table S3
**DISCUSSION**			
Discussion	23a	Provide a general interpretation of the results in the context of other evidence.	Discussion
	23b	Discuss any limitations of the evidence included in the review.	Discussion – Strengths and limitations
	23c	Discuss any limitations of the review processes used.	Discussion – Strengths and limitations
	23d	Discuss implications of the results for practice, policy, and future research.	Discussion
**OTHER INFORMATION**			
Registration and protocol	24a	Provide registration information for the review, including register name and registration number, or state that the review was not registered.	Methods – Protocol registration; Declarations

PRISMA 2020 Checklist

Table S1. PRISMA Checklist			
**Section and Topic**	**Item #**	**Checklist item**	**Location where item is reported**
	24b	Indicate where the review protocol can be accessed, or state that a protocol was not prepared.	Declarations – PROSPERO ID and URL
	24c	Describe and explain any amendments to information provided at registration or in the protocol.	Not applicable (no amendments)
Support	25	Describe sources of financial or non-financial support for the review, and the role of the funders or sponsors in the review.	Declarations – Funding
Competing interests	26	Declare any competing interests of review authors.	Declarations – Competing interests
Availability of data, code and other materials	27	Report which of the following are publicly available and where they can be found: template data collection forms; data extracted from included studies; data used for all analyses; analytic code; any other materials used in the review.	Declarations – Availability of data and materials

From: Page MJ, McKenzie JE, Bossuyt PM, Boutron I, Hoffmann TC, Mulrow CD, et al. The PRISMA 2020 statement: an updated guideline for reporting systematic reviews. BMJ 2021;372:n71. doi: 10.1136/bmj.n71.
This work is licensed under CC BY 4.0. To view a copy of this license, visit https://creativecommons.org/licenses/by/4.0/

Table S2. Search strategies

Database / Platform	Collection / Interface	Exact search string used	Limits / Filters	Initial search	Update
PubMed (NCBI)	MEDLINE / PubMed	Query: ("Respiratory Syncytial Virus Vaccines"[Mesh] OR "Respiratory Syncytial Virus Vaccine" OR "RSV vaccine" OR "RSV vaccination" OR "Maternal RSV vaccination") AND ("Pregnancy"[Mesh] OR "Pregnant Women"[Mesh] OR "Maternal-Fetal Exchange"[Mesh] OR "Maternal Immunization"[tiab] OR "Vaccination in pregnancy" OR "Gestation") AND ("Randomized Controlled Trial"[Publication Type] OR "Randomized Controlled Trials as Topic"[Mesh] OR "RCT" OR "Clinical Trial" OR "Controlled Clinical Trial"[Publication Type]) NOT ("Animals"[Mesh] NOT "Humans"[Mesh])	Humans; no date or language limits at query stage; Phase 3 restriction applied during screening of full texts	Mar 2025	Apr 2025
MEDLINE (Ovid)	MEDLINE (Ovid)	(exp Respiratory Syncytial Virus Vaccines/ OR "Respiratory Syncytial Virus Vaccine".mp. OR RSV vaccine.mp. OR RSV vaccination.mp. OR Maternal RSV vaccination.mp.) AND (exp Pregnancy/ OR exp Pregnant Women/ OR exp Maternal-Fetal Exchange/ OR Maternal Immunization.mp. OR Vaccination in pregnancy.mp. OR Gestation.mp.) AND (exp Randomized Controlled Trial/ OR Randomized Controlled Trials as Topic/ OR RCT.mp. OR Clinical Trial.mp. OR Controlled Clinical Trial/) NOT (exp Animals/ NOT Humans/)	Humans; no date or language limits; Phase 3 restriction during screening	Mar 2025	Apr 2025
Scopus (Elsevier)	Scopus	(TITLE-ABS-KEY("Respiratory Syncytial Virus Vaccine" OR "RSV vaccine" OR "RSV vaccination" OR "Maternal RSV vaccination")) AND (TITLE-ABS-KEY("pregnancy" OR "pregnant women" OR "maternal immunization" OR "vaccination in pregnancy" OR "gestation")) AND (TITLE-ABS-KEY("randomized controlled trial" OR "clinical trial" OR "controlled clinical trial" OR "RCT")) AND NOT (TITLE-ABS-KEY("animals") AND NOT TITLE-ABS-KEY("humans"))	Humans; no date or language limits; Phase 3 restriction during screening	Mar 2025	Apr 2025
Google Scholar	Google Scholar	allintitle: ("Respiratory Syncytial Virus Vaccine" OR "RSV vaccine" OR "RSV vaccination" OR "Maternal RSV vaccination") ("pregnancy" OR "pregnant women" OR "maternal immunization" OR "vaccination in pregnancy" OR "gestation") ("randomized controlled trial" OR "RCT" OR "clinical trial")	No filters available; Phase 3 restriction and human studies applied manually during screening	Mar 2025	Apr 2025

PRISMA flow diagram

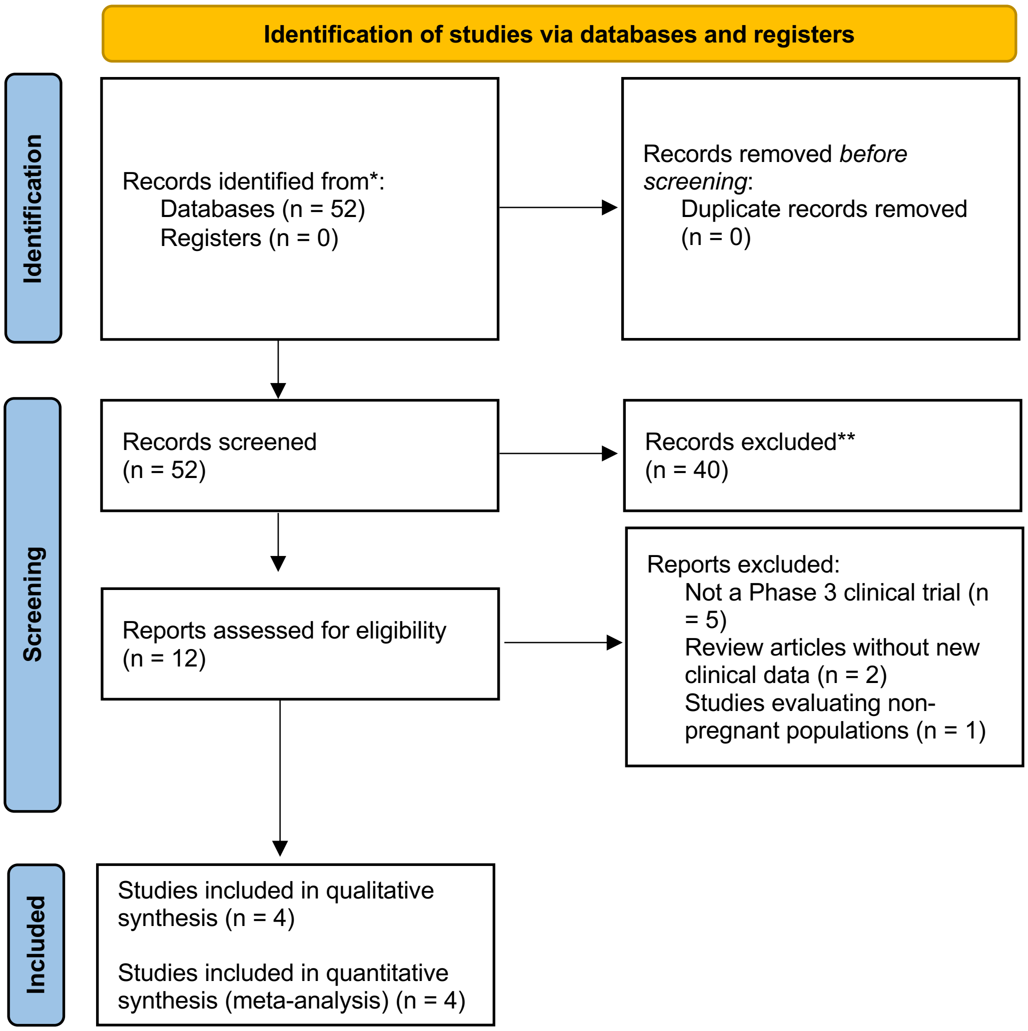

Figure S1. PRISMA flow diagram.

*Consider, if feasible to do so, reporting the number of records identified from each database or register searched (rather than the total number across all databases/registers).

**If automation tools were used, indicate how many records were excluded by a human and how many were excluded by automation tools.

**Table S3.** Risk of Bias Assessment of Included Phase 3 Randomized Controlled Trials (RoB-2 Tool).						
Trial (First Author, Year)	Randomization Process	Deviations from Intended Interventions	Missing Outcome Data	Measurement of the Outcome	Selection of the Reported Result	Overall Risk of Bias
Kampmann, 2023 (MATISSE, Pfizer)	Low risk – Centralized randomization with adequate allocation concealment; baseline characteristics well balanced.	Low risk – Double-blind design with identical vaccine and placebo; adherence closely monitored.	Low risk – Attrition was minimal and reasons for exclusions were well documented.	Low risk – Outcomes assessed through RT-PCR confirmation and adjudication by an independent endpoint committee.	Low risk – All prespecified primary and secondary outcomes were reported according to the protocol.	Low
Madhi, 2020 (Prepare, Novavax)	Low risk – Randomization in a 2:1 ratio with adequate concealment; baseline comparability ensured.	Low risk – Observer-blind design, interventions indistinguishable.	Low risk – Losses to follow-up were limited and balanced between groups; per-protocol and ITT analyses were prespecified.	Low risk – RSV events defined by standardized criteria and confirmed by central laboratory testing.	Low risk – Outcomes consistent with protocol and registry.	Low
Dieussaert, 2024 (RSVPreF3-Mat, GSK)	Low risk – Randomization in a 2:1 ratio, multicenter trial with robust concealment.	Low risk – Investigators, participants, and assessors blinded to allocation; procedures uniformly applied.	Low risk – Attrition moderate but explained; safety and ITT populations clearly reported.	Low risk – RSV outcomes laboratory confirmed, and safety monitored by an independent DSMB.	Some concerns – Early termination due to an imbalance in preterm birth led to protocol amendments and descriptive analyses, raising potential risk of selective reporting.	Some concerns
Simões, 2025 (Final MATISSE analysis, Pfizer)	Low risk – Randomization procedures identical to main MATISSE trial; allocation concealment ensured.	Low risk – Double-blind design with indistinguishable placebo; adherence confirmed.	Low risk – Very low attrition with full follow-up of maternal–infant pairs.	Low risk – Outcomes defined a priori, confirmed by RT-PCR, and adjudicated by independent committees.	Low risk – Prespecified outcomes reported in line with trial protocol.	Low

**Table S4.** Number Needed to Vaccinate (NNV) Derived from Meta-Analysis of Maternal RSV Vaccination Trials		
Outcome	RD (95% CI)	NNV (95% CI)
Any RSV	–0.01 (–0.02 to –0.00)	85 (54–201)
Severe RSV	–0.01 (–0.01 to –0.00)	127 (78–331)
RSV: *Respiratory syncytial virus*; RD: risk differences		

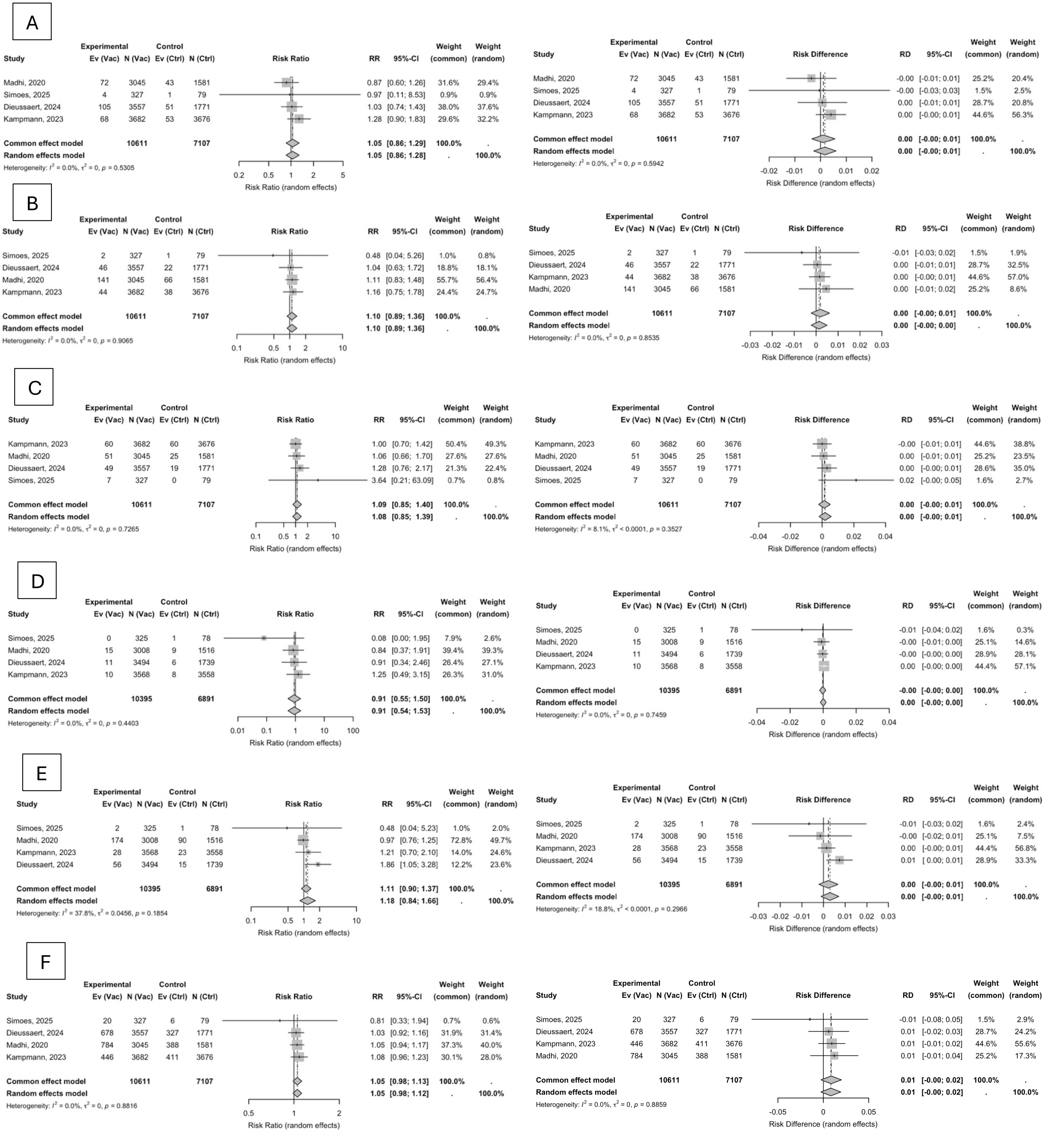

Figure S2. Forest plots of maternal and neonatal safety outcomes following maternal RSV vaccination. Each panel shows pooled risk ratios (RR) and risk differences (RD) with 95% confidence intervals: (A) preeclampsia, (B) gestational hypertension, (C) adverse cardiac events, (D) stillbirth or fetal death, (E) preterm birth, and (F) composite adverse effects (pregnancy, puerperium, perinatal). Squares represent individual trial estimates with size proportional to study weight; horizontal lines indicate 95% confidence intervals; and diamonds represent pooled estimates under a random-effects model.

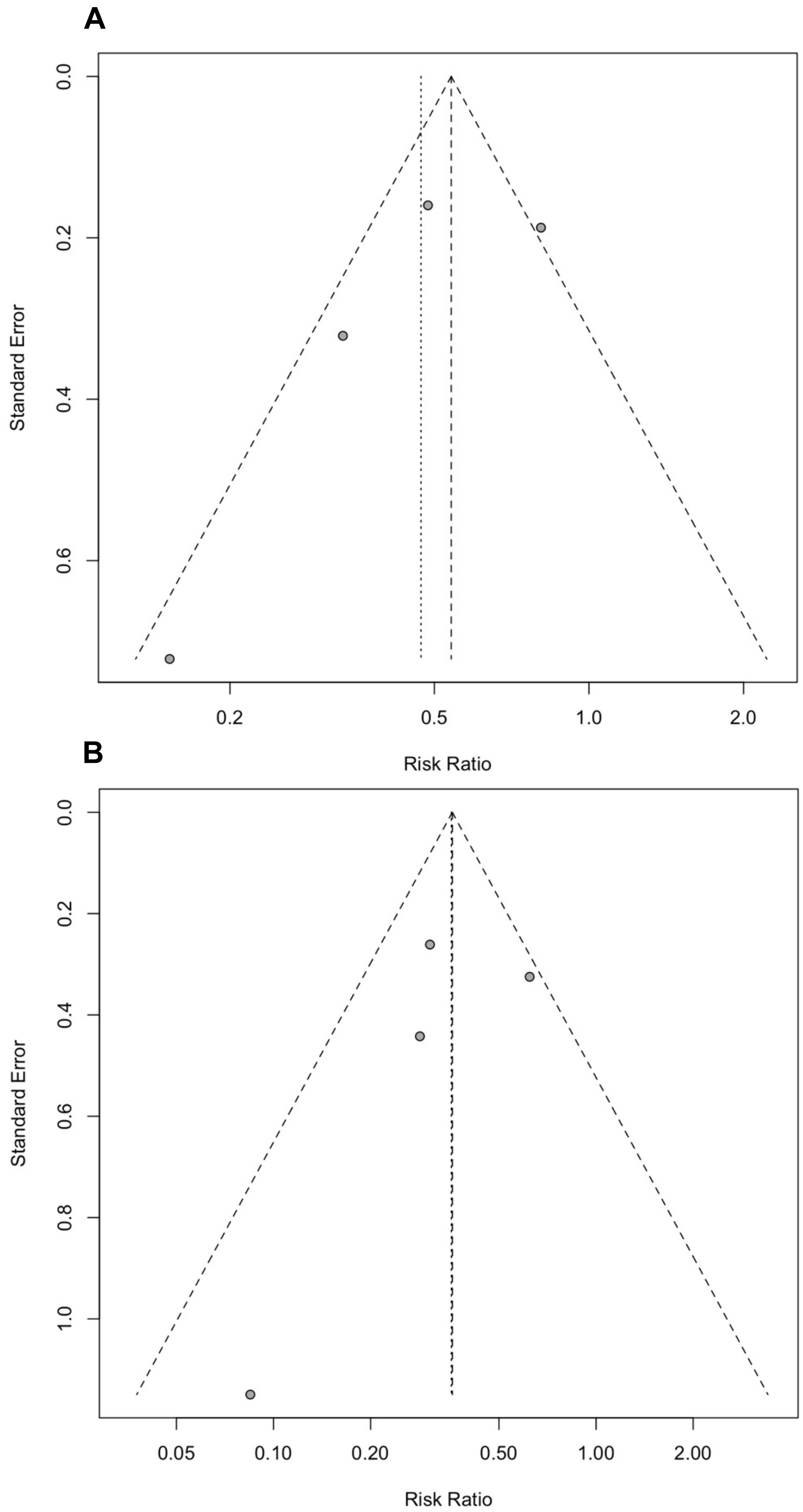

Figure S3. Funnel plots and publication bias assessment for RSV vaccination outcomes.
(A) Funnel plot for any RSV infection
(B) Funnel plot for severe RSV infection
Visual inspection of both plots shows no evidence of asymmetry. Egger's regression test confirmed the absence of publication bias (any RSV infection: p = 0.42; severe RSV infection: p = 0.37).

**Table S5.** GRADE Evidence Profile — Maternal RSV Vaccination vs Placebo								
Outcome (follow-up)	Studies (participants)	Risk of bias	Inconsistency	Indirectness	Imprecision	Publication bias	Overall certainty (GRADE)	Key findings & rationale
Any medically attended RSV infection/LRTI in infants (≤90–180 days)	4 RCTs (≈17,000+ mother–infant pairs)	Not serious	Not serious	Not serious	Serious	Not detected	Moderate (⊕⊕⊕◯)	Directionally consistent protection across trials; downgraded one level for imprecision because at least one trial reported wide CIs including little-to-no effect for the "any RSV" endpoint (e.g., PREPARE primary endpoint and MATISSE 90-day "any RSV" analysis).
Severe RSV disease in infants (≤90–180 days)	3–4 RCTs (same population)	Not serious	Not serious	Not serious	Not serious	Not detected	High (⊕⊕⊕⊕)	Robust and consistent efficacy with narrow CIs around large effects in MATISSE (interim and final) and supportive estimates in GSK; no downgrades.
Preterm birth (<37 weeks)	4 RCTs	Some concerns*	Serious	Not serious	Not serious	Not detected	Moderate (⊕⊕⊕◯)	Imbalance (higher risk) observed in GSK (RR≈1.37, 95% CI 1.08–1.74) while Pfizer/Novavax showed no increase; overall downgraded for inconsistency across trials and early stopping in GSK.
Preeclampsia	3–4 RCTs	Not serious	Not serious	Not serious	Serious	Not detected	Moderate (⊕⊕⊕◯)	No signal of harm; low event rates and wide CIs → downgrade for imprecision.
Gestational hypertension	3–4 RCTs	Not serious	Not serious	Not serious	Serious	Not detected	Moderate (⊕⊕⊕◯)	Rates similar between groups; downgraded for imprecision due to sparse events.

Stillbirth / fetal death	4 RCTs	Not serious	Not serious	Not serious	Serious	Not detected	Moderate (⊕⊕⊕◯)	Very low incidence across trials; wide CIs around small absolute differences → imprecision.
Neonatal death	4 RCTs	Not serious	Not serious	Not serious	Serious	Not detected	Moderate (⊕⊕⊕◯)	Overall rare; GSK reported a small, non-significant imbalance likely mediated by prematurity; downgraded for imprecision.
Congenital anomalies (major)	3–4 RCTs	Not serious	Not serious	Not serious	Serious	Not detected	Moderate (⊕⊕⊕◯)	No excess risk detected; downgraded for imprecision due to rarity.
Infant SAEs through 6–24 months	2–3 RCTs	Not serious	Not serious	Not serious	Not serious	Not detected	High (⊕⊕⊕⊕)	SAE rates similar by group; large samples and consistent findings; no downgrades.
